# Cerebrovascular glycocalyx damage and microcirculation impairment in patients with temporal lobe epilepsy

**DOI:** 10.1177/0271678X231179413

**Published:** 2023-05-26

**Authors:** Rick HGJ van Lanen, Roel HL Haeren, Julie Staals, Jim TA Dings, Olaf EMG Schijns, Govert Hoogland, Sander MJ van Kuijk, Dimitris Kapsokalyvas, Marc AMJ van Zandvoort, Hans Vink, Kim Rijkers

**Affiliations:** 1 Department of Neurosurgery, Maastricht University Medical Centre, Maastricht, The Netherlands; 2 School for Mental Health and Neuroscience (MHeNs), Maastricht University, Maastricht, The Netherlands; 3 Department of Neurology, CARIM School for Cardiovascular Diseases, Maastricht University Medical Centre, Maastricht, The Netherlands; 4 Academic Centre for Epileptology, Maastricht University Medical Centre and Kempenhaeghe, Maastricht/Heeze, The Netherlands; 5 Department of Clinical Epidemiology and Medical Technology Assessment, Maastricht University Medical Centre, Maastricht, The Netherlands; 6 Department of Genetics & Cell Biology, 5211 CARIM School for Cardiovascular Diseases, Maastricht University, Maastricht, The Netherlands; 7 Interdisciplinary Center for Clinical Research (IZKF), University Hospital RWTH Aachen, Aachen, Germany; 8 Institute for Molecular Cardiovascular Research IMCAR, Universitätsklinikum, Aachen University, Aachen, Germany; 9 Department of Physiology, Maastricht University, Maastricht, The Netherlands

**Keywords:** Blood-brain barrier, capillary recruitment, glycocalyx, microcirculation, temporal lobe epilepsy

## Abstract

Temporal lobe epilepsy (TLE) is increasingly associated with blood-brain barrier dysfunction and microvascular alterations, yet the pathophysiological link is missing. An important barrier function is exerted by the glycocalyx, a gel-like layer coating the endothelium. To explore such associations, we used intraoperative videomicroscopy to quantify glycocalyx and microcirculation properties of the neocortex and hippocampus of 15 patients undergoing resective brain surgery as treatment for drug-resistant TLE, and 15 non-epileptic controls. Fluorescent lectin staining of neocortex and hippocampal tissue was used for blood vessel surface area quantification. Neocortical perfused boundary region, the thickness of the glycocalyx’ impaired layer, was higher in patients (2.64 ± 0.52 µm) compared to controls (1.31 ± 0.29 µm), *P < *0.01, indicative of reduced glycocalyx integrity in patients. Moreover, erythrocyte flow velocity analysis revealed an impaired ability of TLE patients to (de-)recruit capillaries in response to changing metabolic demands (*R*^2^ = 0.75, *P < *0.01), indicating failure of neurovascular coupling mechanisms. Blood vessel quantification comparison between intraoperative measurements and resected tissue showed strong correlation (*R*^2^ = 0.94, *P < *0.01). This is the first report on *in vivo* assessment of glycocalyx and microcirculation properties in TLE patients, confirming the pivotal role of cerebrovascular changes. Further assessment of the cerebral microcirculation in relation to epileptogenesis might open avenues for new therapeutic targets for drug-resistant epilepsy.

## Introduction

With a prevalence of 4–10 per 1,000 persons, epilepsy is one of the most common neurological disorders, affecting around 60 million people worldwide.^1^ Over the last two decades, the percentage of drug-resistant patients has remained between 30–40%, despite the development of novel antiseizure medication (ASM).^2^ The societal burden of chronic epilepsy is enormous, encompassing around 80% of total epilepsy-related costs. This is particularly relevant for temporal lobe epilepsy (TLE), the most frequent type of focal epilepsy.^3^ In selected cases of drug-resistant focal epilepsy, resective epilepsy surgery is offered as a highly effective treatment option.^4,5^

Currently, only 20–50% of patients with drug-resistant epilepsy are expected to be candidates for resective brain surgery.^6,7^ The discovery of new drugs targeted at specific underlying pathophysiologic mechanisms holds promise for improved treatment for the remaining patients with drug-resistant epilepsy. In this regard, research is increasingly directed at epilepsy-related abnormalities of the cerebral microcirculation. Altered angiogenesis, microvascular density, structural aberrations and dysfunctional physiological processes, such as neurovascular coupling, have been revealed in the brain tissue of patients with TLE.^8,9^ These microvascular alterations lead to the loss of blood-brain barrier (BBB) integrity.^9–12^ Opening of the BBB induces extravasation of proteins like albumin and leucocytes, leading to astrocytic transformation.^12^ Leucocyte extravasation subsequently leads to local neuroinflammatory processes, increased neuronal excitability, and reorganization of neuronal networks, which increase seizure susceptibility and thus contribute to epileptogenesis.^13,14^

The barrier function of the BBB is mainly determined by the endothelium. Endothelial cells are interconnected by tight junctions and adherent junctions to prevent paracellular diffusion.^15,16^ As such, the endothelium forms a continuous cell membrane layer along the cerebral capillaries. Endothelial cells thus restrict and actively control the passage of substances from the blood to the brain in order to tightly regulate cerebral homeostasis.^15^ As recently highlighted, the endothelial glycocalyx (hereon referred to as ‘glycocalyx’) is also a significant determinant of BBB function.^17,18^ This gel-like layer lines the luminal surface of the endothelium and exerts important barrier properties by limiting leucocyte adhesion and protein extravasation,^19,20^ and has been closely tied to vascular permeability.^21^ Damage to the glycocalyx may initiate BBB dysfunction,^18^ and recent literature continues to report that a disrupted glycocalyx contributes to increased BBB permeability.^21–24^ However, research into the role of the glycocalyx, cerebral microcirculation, and BBB permeability in relation to epilepsy is mainly based on *in vitro* epilepsy models or *ex vivo* evaluation.^9,25,26^ Although these studies contribute to our understanding of microvascular structural abnormalities in epilepsy, their insight into functional microcirculatory characteristics is restricted.

Considering the relationship between previously observed abnormalities in the cerebral microvasculature, a disrupted glycocalyx, increased BBB permeability and the pathophysiology of epilepsy, we hypothesized that patients with focal epilepsy are characterized by altered cerebral microcirculation, and a disrupted glycocalyx in particular. In a recent study, we showed the feasibility of *in vivo* imaging of human cerebral microcirculation and evaluation of its glycocalyx using sidestream dark field (SDF) imaging.^27–29^ Here, we assessed microcirculatory properties—both structural and functional—and glycocalyx dimensions with *in vivo* SDF imaging on the brains of epilepsy patients undergoing resective epilepsy surgery, as well as control patients undergoing brain surgery for other indications. Additionally, we used fluorescent lectin to stain blood vessels of resected neocortical and hippocampal tissue from patients with TLE who had undergone intraoperative SDF imaging, allowing assessment of the relation between *in vivo* and *ex vivo* blood vessel surface area.

## Material and methods

This prospective observational case-control study was approved by the local medical ethical committee (METC azM/UM) and assigned protocol ID: NL51594.068.14. This study was prospectively registered at the International Clinical Trials Registry Platform (ID: NTR5568), and complies with the Declaration of Helsinki and principals of Good Clinical Practice. Patients were included upon obtaining informed consent.

### Selection of study participants

The protocol of this study has been published previously.^27^ In brief, we included patients between 18 and 60 years of age with unilateral drug-resistant temporal lobe epilepsy (TLE) scheduled for resective brain surgery, i.e., anterior temporal lobectomy with amygdalohippocampectomy (further referred to as ‘patients’). The diagnosis of unilateral TLE and eligibility for resective surgery was assessed by a multidisciplinary team after thorough presurgical examinations.

Control individuals consisted of neurosurgical patients between 18 and 60 years of age without a history of epileptic seizures, who underwent a craniotomy for intracranial tumor resection or elective, without previous subarachnoid hemorrhage, aneurysm clipping (further referred to as ‘controls’).

Exclusion criteria included pregnancy, history of established hypertension, diabetes mellitus, hyperlipidemia, stroke or other cardiovascular disease, use of cardiovascular medication, or non-symptomatic signs of cerebral small vessel disease on brain MRI. Additionally, controls were excluded if they had a ‘compressed’ and/or ‘edematous’ cerebral cortex on MRI or during surgery, or a reported history of seizures.

### Demographic and clinical data

We collected the following clinical data: patient characteristics (age, smoking, handedness), medical history (febrile seizures, traumatic brain injury, cerebral infection, familial epilepsy), seizure characteristics (age at debut, type of seizures, seizure frequency, length of seizures, status epilepticus (SE) frequency), and use of antiseizure medication (ASM) and other medication.

### Measurements

Microvascular properties and glycocalyx thickness were assessed intraoperatively at the following time points: (1) cortical measurement after opening of the meninges, allowing a view of the cortex in both patients and controls, (2) hippocampal measurement (only in patients) performed upon removal of the temporal neocortex, allowing a direct view of the lateral hippocampus (images shown in supplementary material 1). In controls, intraoperative measurements were performed as far away from the tumor as possible, as determined by the craniotomy size. At each time point, systolic and diastolic blood pressure, heart rate, pulse oxygen saturation, end-tidal pCO_2_, hemoglobin concentration, and hematocrit were recorded. All subjects had a similar standard protocol for induction of anesthesia using propofol, piritramide, and rocuronium, and were administered 2 grams of cefazoline.

Measurements were performed using a sidestream darkfield (SDF) video microscope (GlycoCheck & Microvascular Health Solutions Inc., Salt Lake City, UT, USA), enclosed in a sterile slipcover. The video microscope consists of a central light guide with a magnifying lens and concentric light emitting diodes. The diodes emit light at a wavelength of 530 nm, which is absorbed by (de)-oxyhemoglobin in erythrocytes. Consequently, erythrocytes appear black on a greyish background. The analysis is based on the principle of the erythrocyte-endothelial exclusion zone. The system measures the variation of the red blood cell (RBC) column’s penetration in the glycocalyx. This variation increases with a damaged or weaker glycocalyx. GlycoCheck software continues to collect videos until ∼3000 microcirculatory vessel segments are successfully included. The complete measurements contain between 10 and 30 videos, depending on the number of vessel segments evaluated in each video. In each vessel segment, the RBC column width (RBCW) is measured, and vessels are automatically grouped into separate diameter classes at 1 µm intervals, ranging from 4 to 25 µm in diameter. This concept has been successfully used and validated in previous studies.^28–30^ Given the novel application site of the SDF video microscope, i.e., the brain’s neocortex and hippocampus, and the effects of the sterile slipcover,^28^ we performed an additional manual assessment to verify that GlycoCheck selected only adequately visualized vessels, and to assess whether the quality of the videos made with the SDF camera were reliable before further analysis (see Supplementary material 2).

### Glycocalyx parameters: perfused boundary region (PBR)

As an indirect measure of glycocalyx integrity, we calculated dynamic perfused boundary region (PBR, µm), which is the outermost luminal part of the glycocalyx that is only slightly permeable to erythrocytes.^17^ The software calculates the dynamic lateral movement of RBCs into the permeable part of the glycocalyx layer. An impaired glycocalyx permits a greater number of RBCs to penetrate into the glycocalyx, which is thus reflected by higher PBR values.^31^ The penetration of RBCs into the glycocalyx is dependent on blood flow velocity and volume. To minimize flow-dependent variability in PBR estimation, we used dynamic PBR values. Details on the acquisition and calculation of PBR have been described previously.^28,29,32^

### Microvascular parameters

The following microvascular and microcirculatory properties were collected *in vivo* intraoperatively:

- Perfused vascular density. Vessels were separated into capillaries (diameter classes 4–7 µm) and vessels (diameter classes 10–25 µm). An absolute measure for perfused vascular density (mm/mm^2^) can be determined from the number of vascular segments containing RBCs multiplied by capillary segment length (each 10 µm). All detected RBC-containing vessel segments (RBC content ≥50%) were automatically counted in the video recordings of each subject and normalized to tissue surface area. As non-perfused vessels (i.e., vessels without RBCs present), as well as vessels not meeting the quality criteria cannot be detected using our methodology, vascular density in this manuscript refers to valid perfused vascular density (hereafter vascular density).
- RBC velocity (V_RBC_). The V_RBC_ was determined for individual vessel segments in an automatic fashion by cross correlation of longitudinal RBC displacement. V_RBC_ was determined by dividing RBC displacement distance by the time between video frames, expressed as µm/s.
- Capillary recruitment. To account for the ability to recruit additional capillaries, capillary recruitment can be estimated by measuring the slope of the relationship between the V_RBC_ of capillaries (diameter classes 4–7 µm) and afferent V_RBC_ (diameter classes 10–25 µm). When the number of perfused blood vessels increases in afferent V_RBC_, the accompanying increase in capillary V_RBC_ will be less than proportional (i.e., the regression slope will be <1) and capillary recruitment can be defined as 1 – slope(capillary V_RBC_, afferent V_RBC_). For example, if the amount of perfused capillaries doubles when afferent V_RBC_ doubles, the slope(capillary V_RBC_, afferent V_RBC_) will be 0, thus capillary recruitment = 1 – slope(0) = 1 = 100%. In contrast, if the number of perfused capillaries does not change when afferent V_RBC_ increases twofold, capillary V_RBC_ is expected to also change proportionally by twofold, and the slope(capillary V_RBC_, afferent V_RBC_) will be 1, thus capillary recruitment = 1 – slope(1) = 0 = 0%. A capillary recruitment of 78% has been described in healthy controls.^29^
- Cerebral Blood Surface Area (CBSA, µm^2^/µm^2^). The CBSA can be calculated by determining the number of vascular segments multiplied by the vascular segment length (i.e., vascular density; mm/mm^2^) and segment-specific capillary diameter (µm). CBSA can be used for correlation with *ex vivo* blood vessel staining quantification.

Details on the acquisition and calculation of all outcome measures have been described previously.^28,29^

### Histopathological and blood vessel staining analysis

Resected tissue was routinely examined by a neuropathologist to establish a histopathological diagnosis in patients and controls. In patients, histopathological examination was used to identify hippocampal sclerosis (HS) and focal cortical dysplasia (FCD) according to ILAE classifications.^33–35^

To verify *in vivo* blood vessel quantification as measured using SDF video microscopy, we analyzed retrospectively resected neocortex and hippocampus tissue using fluorescent lectin to stain blood vessels. Both neocortex and hippocampus tissue samples were collected from 10 patients (P2, P3, P5, P7, P8, P9, P10, P11, P12, P13). For each patient, one neocortex and one hippocampus section (5 µm in thickness) were used to calculate total Blood vessel Stained Surface Area (BSSA) by dividing total vessel area (µm^2^) by total sample area (µm^2^). For details on staining, visualization and quantification see Supplementary material 3.^36,37^

### Statistical analysis

Based on the power size calculation described in the protocol paper,^27^ we aimed to find a minimal difference in glycocalyx thickness of 12% as clinically significant, with an expected standard deviation (SD) of 15%.^31,38^ With an α of 0.05 and 1−β of 0.80, a sample size of 13 participants per group was calculated using the 1-sample Z-test. We aimed to include 15 subjects per group to overcome a possible drop-out of two subjects per group.

All data was tested for distribution using the Shapiro-Wilk test. Data are presented as mean and SD when normally distributed, or as median and IQR when non-normally distributed. Data between groups are compared using the independent samples t-test or the Mann-Whitney U-test, as appropriate. Correlation between cortical and hippocampal glycocalyx PBR is calculated by Pearson’s or Spearman’s correlation coefficient, when data are normally or non-normally distributed, respectively. Cortical and hippocampal microvascular properties and glycocalyx PBR results are correlated to seizure characteristics, epilepsy risk factors, ASM usage and histopathological diagnosis according to ILAE classification^35^ using Pearson’s or Spearman’s correlation coefficient or univariable and multivariable regression analysis, as appropriate. The association between demographic and clinical parameters and microvascular properties and glycocalyx PBR results are quantified using univariable and multivariable regression analysis. Cortical and hippocampal CBSA and fluorescent lectin blood vessel staining counts are correlated using regression analysis. Statistical analyses were performed with IBM SPSS software version 27 or higher.

## Results

### Patient and control characteristics

Between 2016 and 2021, our prospective study included fifteen patients with TLE and fifteen controls. Mean age in the groups was 39.9 (SD 10.8) and 49.1 (SD 10.0), respectively, (*P *= 0.019). No differences in sex ratio, mean BMI, or smoking rates were noted. Subject characteristics are shown in Table 1.

**Table 1.** Demographic characteristics of the study population.

		Patients(n = 15)	Controls(n = 15)	p-value
Mean age (SD), years		39.9 (10.8)	49.1 (10.0)	.019
[range]		[23–55]	[23–59]	
Sex (%)	Male	5 (33.3)	8 (46.7)	.462
Body mass index (SD), kg/m^2^		26.0 (3.4)	28.9 (6.4)	.126
Smoking (%)	Yes	2 (13.3)	3 (20.0)	1.000
Ictal onset zone of epilepsy	Neocortical TLE	5	–	
	Mesial TLE	10	–	
Indication for surgery	Vascular	–	5	
	Oncology	–	10	

In patients with TLE, mean age at onset of epilepsy was 21.0 years (SD 11.2), and mean time since onset of epilepsy was 18.9 years (SD 13.5). All subjects in this group used ASM (average number of ASMs was 2.13 (SD 0.88)). Histopathological examination showed hippocampal sclerosis (HS) in eight patients (ILAE type 1 in six patients; type 2 in one patient; and type 3 in one patient), six were negative, and HS was undetermined in one patient. No focal cortical dysplasia was found. In controls, five subjects underwent surgery for elective clipping of an intracranial aneurysm, and ten underwent surgery for resection of an intracranial tumor. For comprehensive information on epilepsy characteristics, medical history, MRI, and histopathological findings of patients, as well as characteristics of controls, see Supplementary material 4: tables 1 and 2.

### Measurements

A total of 27 cortical (13 controls and 14 patients) and 14 hippocampal measurements were performed. Some measurements could not be completed due to air bubbles between the lens of the camera and the brain’s surface. Despite attempts to solve this artifact by substituting the camera tip and replacing the slipcover, we could not recover a good signal-to-noise measurement. Cortical measurements in patients were performed on the surface of the temporal neocortex, either on the superior or middle temporal gyrus. The location of the cortical measurements in the controls depended on surgical indication. In controls with a vascular indication, measurements were performed on the superior temporal gyrus (*n *= 5); in controls with an oncological indication for surgery, location depended on tumor site and exposure, and was either superior frontal gyrus (*n *= 3), middle frontal gyrus (*n *= 3), superior parietal lobule (*n *= 1), inferior parietal lobule (*n *= 1), or superior temporal gyrus (*n *= 2). Measurements were performed as far away from the tumor as possible. No adverse events were recorded during the duration of this study.

Average recording time (7.2 minutes) was similar between patients and controls, and between cortical and hippocampal measurements. Cortical measurements included on average 1043.5 valid vessel segments in 16.3 videos for patients, and 1368.6 valid vessel segments in 22.2 videos for controls. Hippocampal measurements averaged 1304.8 valid vessel segments in 21.1 videos. Intraoperative systolic and diastolic blood pressure, heart rate, pulse oxygen saturation, end-tidal carbon dioxide, hemoglobin concentration, and hematocrit were comparable between patients and controls (see Supplementary material 4: table 3).

### Perfused boundary region

**Figure 1.**
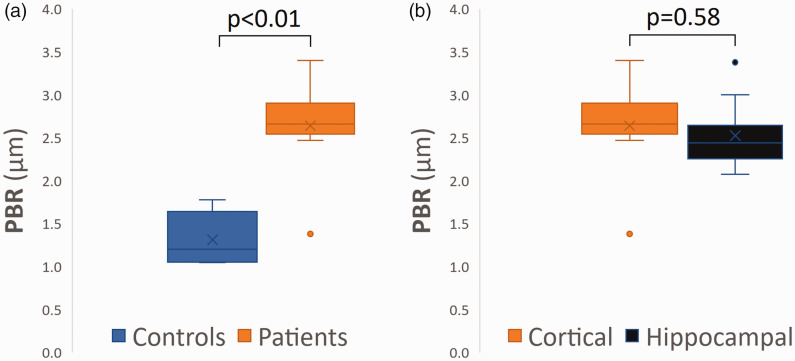
Perfused Boundary Region (PBR) values in µm. (a) comparison of cortical PBR values between patients and controls. The significantly higher PBR values in patients are indicative of a more damaged glycocalyx and (b) comparison of cortical and hippocampal PBR values in patients.

Mean cortical PBR was higher in patients (2.64 µm; SD 0.52) than in controls (1.31 µm; SD 0.29), *P < *0.01, suggesting a damaged glycocalyx in patients (Figure 1(a)). In patients, the PBR in the blood vessels of the neocortex was similar to that in the hippocampus (2.53 µm; SD 0.39), *P *= 0.58 (Figure 1(b)). Patients with HS (HS+) and without (HS−) showed comparable mean PBR values of hippocampal vessels (2.56 µm; SD 0.42 vs. 2.50 µm; SD 0.47, respectively), *P *= 0.86.

### Vascular density

**Figure 2.**
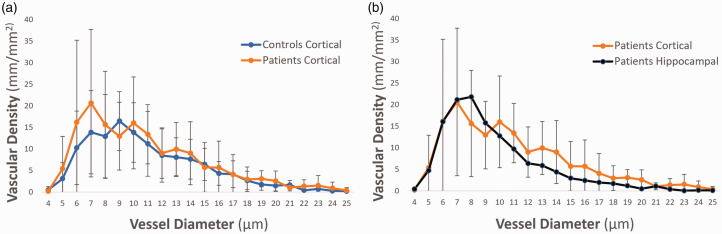
Vascular density per vessel diameter class. (a) cortical vascular density of patients and controls; there were no significant differences detected in any of the diameter classes, albeit a trend can be observed for diameter classes 5 µm (+74%), 6 µm (+58%), and 7 µm (+49%) in patients compared to controls and (b) comparison of patient cortical and patient hippocampal vascular density; no significant difference was observed.

Vascular density, compared in a diameter-class-wise fashion from 4–25 µm (Figure 2(a)), was similar between patients and controls (7.19 mm/mm^2^; SD 4.00 vs. 5.95 mm/mm^2^; SD 2.62), *P *= 0.43. However, a trend towards an increase in vascular density in patients was noted for the capillary diameter classes 5, 6 and 7 µm, which showed an increase of +74%, +58% and +49% in density, respectively (*P *= 0.35). In patients, mean hippocampal and cortical vascular densities were comparable, showing higher vascular density in vessel diameter classes 5 to 8 µm, and lower for the remaining diameter classes 8 to 25 µm (Figure 2(b)). Hippocampal vascular density did not differ between HS+ and HS− patients (6.73 mm/mm^2^; SD 3.49 vs. 5.57 mm/mm^2^; SD 2.50, respectively), *P *= 0.59.

### Red blood cell velocity and capillary recruitment capacity

**Figure 3.**
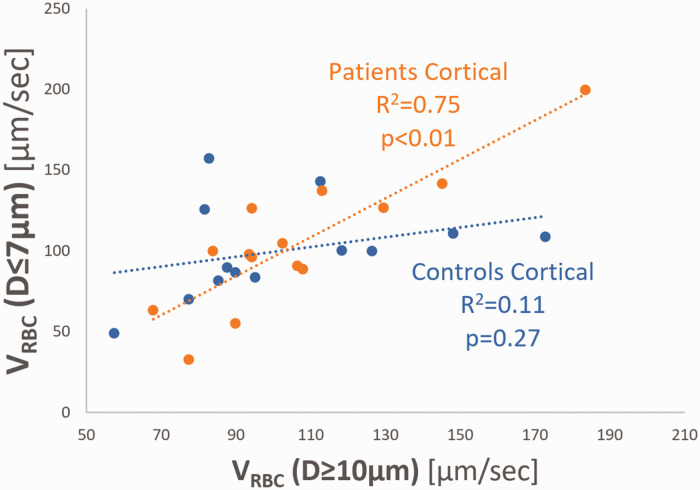
Scatter dot plots and linear regression of cortical V_RBC_ in capillaries (diameter classes 4–7 µm) plotted against cortical V_RBC_ in feed vessels (diameter classes 10–25 µm) of patients and controls. A strong dependency between cortical capillaries and feed vessels was found only in patients.

**Figure 4.**
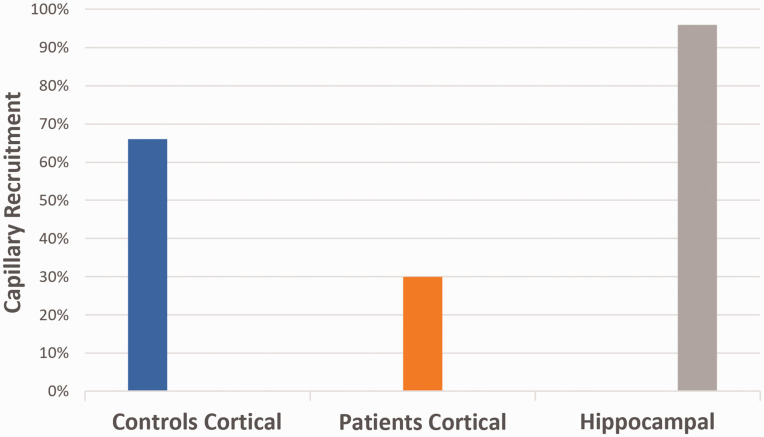
Capillary recruitment capacity of the microcirculation in patients and controls. A clear decrease in capillary recruitment capacity was found in the cortical blood vessels of patients compared to controls. Hippocampal recruitment capacity was high (96%), though no control data is available.

We plotted capillary red blood cell (RBC) velocity, V_RBC_ (vessel diameter classes 4–7 µm, D ≤ 7 µm) as a function of afferent vessel V_RBC_ (vessel diameter 10–25 µm, D ≥ 10 µm). This analysis revealed a strong dependency between capillary V_RBC_ (D ≤ 7 µm) and afferent vessel V_RBC_ (D ≥ 10 µm) in the cortex of patients (*R*^2^ = 0.75, *P < *0.01), indicating impaired capillary (de)recruitment in the cerebral cortex of patients (Figure 3). In contrast, capillary V_RBC_ was relatively stable in controls, showing no dependency between capillary V_RBC_ and afferent vessel V_RBC_ (*R*^2^ = 0.13, *P *= 0.34). Next, we calculated capillary recruitment capacity (Figure 4) as 1 – slope(capillary V_RBC_, afferent V_RBC_). Cortical capillary recruitment capacity was calculated at 30% in patients, and 66% in controls. Capillary recruitment capacity in the hippocampal microcirculation was calculated at 96%.

### Cerebral blood vessel surface area

**Figure 5.**
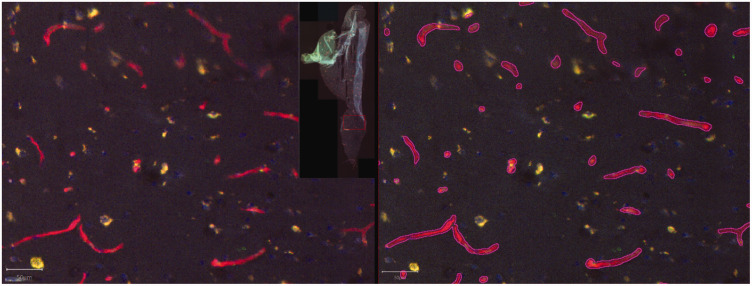
Example of a visualized Ulex Europaeus Agglutinin I (UEA-I) lectin-stained neocortical sample. Left before and right after automated annotation of vessels (purple delineations). Inset image on the left shows the entire slice and magnified area (red box).

**Figure 6.**
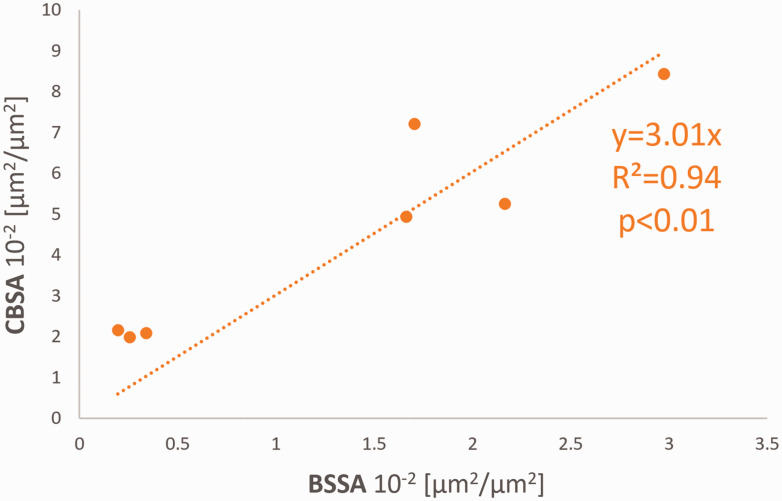
Scatter dot plot and linear regression of Cerebral Blood Surface Area (CBSA) and Blood vessel Stained Surface Area (BSSA). Quantification of blood vessel results are in 10^−2^ µm^2^/µm^2^. Linear regression showing a good fit (*R^2^* = 0.94). The function describing the relation between these two measurements shows a 3.01 times higher blood vessel surface area count.

We used Cerebral Blood Surface Area (CBSA) as a reference to compare *in vivo* SDF videomicroscopy data on vessel density with *ex vivo* fluorescence Blood vessel Stained Surface Area (BSSA). The quality of lectin staining was insufficient for quantification in five patients (P2, P3, P8, P11, P12), and one patient (P5) had missing intraoperative data, therefore these patients were excluded for comparison of CBSA and BSSA. Stained resected tissue (example in Figure 5) that was used to validate *in vivo* data included neocortex tissue of three patients (P9, P10, P13) and hippocampus tissue of four patients (P7, P9, P10, P13). Results of *in vivo* CBSA were compared to *ex vivo* BSSA from the same patient. CBSA for *in vivo* measurements averaged 4.58 10^−2^ µm^2^/µm^2^ (SD 2.62), while BSSA in the stained samples averaged 1.33 10^−2^ µm^2^/µm^2^ (SD 1.09). Analysis of the correlation between these two parameters showed a linear regression of *R^2^* = 0.94 (*P < *0.01; Figure 6). The equation representing this correlation, y = 3.01x , indicates that the *in vivo* CBSA measurements (total blood vessel surface area per image) are three times higher than measurements obtained by *ex vivo* BSSA. Additionally, we found a significant correlation between blood vessel density and PBR, for both in vivo (Pearson correlation r for CBSA and PBR: 0.78, P = 0.040) and ex vivo (Pearson correlation r for BSSA and PBR: 0.76, P = 0.046). Regression analysis for these parameters shows that with increasing vascular density, PBR values also increase (y = 0.86*10^−2^x + 0.12 × 10^−2^, R^2^ = 0.53).

### Other characteristics

We found no association of cortical PBR, hippocampal PBR or microvascular properties with age, smoking, BMI, medical history, seizure characteristics, or use of medication in univariable or multivariable (regression) analyses. In patients, a shorter seizure duration was associated with increasing hippocampal PBR (*P < *0.01), however there was no such correlation for cortical PBR values.

## Discussion

This is the first study to report *in vivo-*assessed cerebrovascular glycocalyx integrity and microcirculation properties of patients with TLE. In this prospective clinical study with a predefined and published protocol, power analysis, and statistical plan,^27^ we show that patients with drug-resistant TLE have altered cerebral microcirculatory properties—both structural and functional—in comparison to controls. Specifically, the cortical microvasculature of patients is characterized by reduced glycocalyx integrity compared to that of the controls. Furthermore, we found impaired blood flow control and capillary recruitment capacity in the cortical microcirculation of patients. To verify our *in vivo* results, we compared the *in vivo* blood vessel surface area with *ex vivo* blood vessel staining quantification in patients using the same tissue, which showed a strong correlation between the two assessments.

### Cerebral endothelial glycocalyx damage in epilepsy

To date, the role of glycocalyx alterations in epilepsy has not been established. We provide the first evidence that TLE is accompanied by damage to the glycocalyx by showing a clear increase in PBR values in the neocortical vasculature in patients with TLE. This epilepsy-associated damaged glycocalyx may contribute to the development of TLE, in addition to being the result of seizures. We discuss our results on glycocalyx integrity in light of both hypotheses. Since the glycocalyx plays a pivotal role in vascular permeability, damage to the glycocalyx increases the extravasation of water, proteins, and other molecules into the brain tissue.^18,21,39^ This allows leukocytes and large proteins to enter the brain parenchyma,^18,40^ leading to altered brain homeostasis and astrocyte functioning, as well as initiating neuroinflammatory processes.^41^ After glycocalyx degradation, the adhesion molecules ICAM-1 and VCAM-1 are exposed, promoting leukocyte adhesion to, and transmigration across, the BBB.^23,42^ The subsequent impairment of neuro-vascular coupling leads to epileptogenesis and seizures.^43,44^ Upregulation of proinflammatory cytokines as a response to leakage of leucocytes further contributes to the corresponding increased neuronal excitability and the production of matrix metalloproteinases, thus damaging the brain tissue.^23,42^ Indeed, there is increasing evidence for the hypothesis that BBB disruption contributes to the development of epilepsy.^8,16,22,35,43,45^ Considering the damaged glycocalyx that results from seizures, this may be related to hemodynamic alterations that are characteristic of seizures.^9^ In case of seizures, the related extreme hemodynamic circumstances such as sinus tachycardia and hypertension result in increased blood flow shear force to the wall of cerebral blood vessels,^46^ ultimately degrading the glycocalyx.^47^ Using a rat blood vessel model, Lyu et al. found enzymatical degradation of the glycocalyx under changes of blood flow shear force.^48^ Additionally, increased BBB permeability has been described following a seizure, which in turn leads to BBB dysfunction and progression of epileptogenesis.^43,44^ The measured epilepsy-associated glycocalyx damage could therefore potentially serve as a marker of BBB dysfunction.^18,21,24^

### Impaired cerebral microcirculation in epilepsy

In this study, the tendency for higher capillary density in patients, combined with impaired cortical recruitment capacity, indicate a higher metabolic demand in patients' tissues along with impaired metabolic flow control. Together this suggests impairments in crucial cerebrovascular regulatory mechanisms. In physiological conditions, pial arteries and parenchymal arterioles continuously adapt the local cerebral blood flow to systemic hemodynamic changes, i.e., autoregulation,^49^ and alterations in neuronal activity and its accessory metabolism, i.e., neurovascular coupling.^50,51^ To accommodate the increased blood flow and metabolic demand, previously closed capillaries can be opened, i.e., capillary recruitment. If capillary recruitment is impaired, i.e., the increase in perfused capillary density does not sufficiently accommodate the rise in cerebral blood flow, RBC velocity within capillaries will increase instead, thereby reducing capillary transit time. To maintain sufficient capillary oxygen extraction, capillary RBC velocities need to remain relatively limited with sufficient capillary transit time.^52^ In this study, we found that patients with TLE were not able to maintain constant capillary RBC velocities. Specifically, we observed changes in capillary RBC velocities proportional to RBC velocity changes in feeding vessels. These findings illustrate that the number of perfused capillaries in TLE patients is essentially fixed and insensitive to local variations in tissue metabolic demand or cerebral blood flow. Such failure of capillary recruitment and neurovascular coupling was not found in controls, where capillary RBC velocities seemed to be independent of the associated RBC velocity in the feeding vessels, in line with previous observations.^29,53^ This indicates that (de)recruitment of capillaries was associated with changes in feeding vessels’ blood flow in controls. The impairment of cortical cerebrovascular regulatory mechanisms is supported by a mismatch in flow dependency of PBR in feed blood vessels (Supplementary material 5). Specifically, controls show low PBR at low flow sites and high PBR at high flow sites, indicating that metabolic flow control is intact, and that low metabolic sites have low blood flow and low PBR due to the low metabolic challenge of the microvascular glycocalyx. In contrast, patients show high PBR at low flow sites, indicating impaired metabolic flow control. One solution for adapting to the noted failure of capillary recruitment in TLE patients is the formation of new capillaries, i.e., angiogenesis, which leads to an increase in capillary density. In our study, we indeed found a trend towards an increased number of capillaries in the cortex of patients, although this was insufficient to compensate. Furthermore, hemodynamic changes during seizures lead to the simultaneous and severe challenge of autoregulatory responses of cerebral arteries, as well as to an increase in metabolic burden of the involved brain areas,^23^ thereby challenging neurovascular coupling mechanisms.^54^ Additionally, neurovascular decoupling and microcirculatory mismatch are possibly exaggerated by hampered shear stress-induced nitric oxide production by an impaired glycocalyx, leading to excessive vasoconstriction of parenchymal arterioles.^55^ This neurovascular decoupling results in an unintended reduced cerebral blood flow in the downstream capillary bed, and hypoxia.^8,56^ Epileptogenesis is also linked to a misbalance in angiogenesis homeostasis.^9^ Neuronal activity and reactive astrocytes may form an alternative pathway for induced angiogenesis, creating a misbalance between barriergenesis and endothelial cell proliferation that leads to the formation of functionally mature, yet leaky vessels. This would result in disturbed angiogenesis, abnormal microvascular morphology and microvascular density, and BBB dysfunction.^9,42^ Together, these microvascular changes and neurovascular decoupling disturb the parenchymal homeostasis in patients with epilepsy, which in itself forms a substrate for facilitating epileptogenesis and seizures.^8^

Our finding of increased PBR in TLE reflect a damaged and dysfunctional glycocalyx, but our results have not proven that the glycocalyx layer as a whole is thinner. This would require assessment and imaging of the ‘intact’ cerebrovascular glycocalyx as a whole. However, measurement methods to assess or image the glycocalyx layer directly, lead to alterations of the glycocalyx layer due to the measurement method itself, leading to shedding.^17^ Especially measurement of glycocalyx thickness in tissue samples leads to results that cannot be extrapolated to the in vivo situation, as the glycocalyx layer directly starts breaking down and shedding when blood vessel perfusion is halted.^24^ For this reason has the cerebrovascular glycocalyx not been measured in humans—only using rodent models.^57,58^ For example, Yoon et al. measured the glycocalyx thickness *in vivo* in cerebral pial arteries (1.15 µm; SD 0.03) and capillaries (0.42 µm; SD 0.02) of mice.^57^ In a rat model, damage to the endothelial glycocalyx due to asphyxia, complicated infection, iatrogenic excessive fluid infusion, and hyperglycemia resulted in severe glycocalyx degradation and increased BBB permeability.^23^ Additionally, in a mouse model of status epilepticus (SE), glycocalyx degradation was the first step in the pathophysiological process of brain injury after SE.^59^ These results showed that glycocalyx damage aggravated brain injury, while protection of the glycocalyx reduced BBB dysfunction and alleviated brain damage.^23,59^ Studies addressing the glycocalyx in humans have only used sublingual measurements and focused on other pathologies, especially sepsis and neurodegenerative diseases.^29,53^ *In vivo* assessment of the cerebral microcirculation in humans has only been reported in one study, which used SDF imaging to assess the intraoperative cerebral microcirculation in peritumoral edema.^60^ However, vascular density and microvascular flow were measured with different methods than those used in our study, therefore the results cannot be compared directly. Imaging of the human cerebrovascular microcirculation during vascular neurosurgery has been performed using orthogonal polarizing spectral (OPS) imaging, the preceding video microscopy technique of SDF imaging.^61,62^ Using OPS imaging, diameter changes of arterioles between 15 to 180 µm were observed. Vascular density or red blood cell dynamics were not observed, nor were capillaries included here, as capillaries are typically 4 to 7 µm in diameter.

### Cerebral vascular density in epilepsy

Vascular density in epilepsy has mainly been studied in preclinical epilepsy models using *in vitro* techniques.^9^ Studies assessing cortical vascular density in rodent models found it to be increased, although no exact values were provided.^63,64^ In the hippocampal microcirculation, vascular densities have been reported to be increased, unchanged, and decreased, varying from 80 to 140% of controls.^25,63–65^ In human epilepsy, we found only one study to report vascular density changes in the cortex of two HS+ TLE patients using postmortem whole brains, which were similar to controls.^66^ Postmortem studies on hippocampal tissue showed that hippocampal vascular density is increased in TLE patients.^26,67^ This is in line with our intraoperative vascular density results, where we found increased—albeit not significant—vascular density in the cortex of patients compared to controls, whereas hippocampal and cortical vascular density were similar. Our in vivo vascular density results showed high variance, which might be due to patient characteristics, or in controls due to different recording sites. Based on our histological samples we cannot make an estimation if vessel density is increased, since we used vessel density in histological samples only for comparison with in vivo vessel density results. Additionally, comparison with other vascular density studies is not possibly due to the difference in used staining and counting methods.^9^ Thus, whether cerebral vascular density in epilepsy is increased, decreased or unchanged remains unclear, but our results and previous literature tend towards an increase.^9^

To verify and validate our *in vivo* measurements of CBSA, we compared CBSA with *ex vivo* BSSA lectin staining quantification. This comparison showed a good correlation (*P *= 0.94), confirming and strengthening our *in vivo* results. We used surface area for both *in vivo* and *ex vivo*, as only these measures could be used for comparison, in contrast to blood vessel counts which cannot be extrapolated from the *in vivo* data. We found a three-fold increase between the *ex vivo* and *in vivo* assessment of the blood vessel surface area, as reflected by the equation y = 3.01x. This may be due to the fact that *in vivo* blood vessels are opened and perfused, while under *ex vivo* conditions the vessels are collapsed, thus occupying less surface area. Furthermore, SDF videomicroscopy is able to detect integrative blood vessels to a depth of at least 10 µm,^29^ while slices used for *ex vivo* were maximally 5 µm thick. This again would result in an increase of total measured blood surface area *in vivo* compared to *ex vivo*.

We noticed a significant correlation between blood vessel density, both in- and ex vivo, and PBR. Regression analysis showed that higher vascular density was correlated to higher PBR values. Thus, the more blood vessels are present, the more damaged the glycocalyx in these vessels is. These findings are in line with our previous hypothesis that neuronal and epileptic activity results in increased angiogenesis, leading to an increase of microvascular density, while barriergenesis lacks behind, leading to leaky blood vessels.^9^ A damaged glycocalyx may be a major contributing factor to the increased permeability of these blood vessels.

### Limitations

This study has several limitations. For example, our controls were significantly older than patients. While literature on sublingual glycocalyx measurements have reported increasing PBR values in older patients,^68^ PBR was not correlated with age in our cohort, suggesting that this was not a confounding factor in our data. This may be related to the upper age limit (60 years old) for inclusion in our study, which was selected to limit the effect of background age-related microvascular disease. Next, technical limitations were a factor. All measurements were performed by two investigators experienced with the SDF camera. Nevertheless, cortical measurements of patients and controls sometimes proved difficult due to pressure artefacts, effects of the sterile slipcover, and the pulsating cerebral cortex. Though all measurements lasted until ∼3000 microcirculatory vessel segments were collected, the GlycoCheck built-in quality control excludes incorrect vessel segments only after the measurement, resulting in the loss of a portion of possible data (see Supplementary material 2). Replacing the sterile slipcover for a sterile hardcover tube could potentially improve valid vessel yield in future studies. Additionally, all calculations are ultimately based on the flow properties of RBCs, meaning we could only analyze blood vessels in which a minimum number of RBCs are present and the predefined quality criteria were met.^27,29^ Vessels without RBCs or invalid vascular segments are therefore not detected by the software, which could impact vascular density calculations. Moreover, all of our measurements were performed on patients under general anesthesia. Little is known about the effects of general anesthesia on microcirculatory changes, specifically those related to cerebrovascular microcirculation. Increased sublingual PBR values after surgery have been reported.^69,70^ Although the extent of glycocalyx damage does not seem to be affected by anesthetics, similar standard anesthesia induction using propofol was applied for all patients of our study.^71^ Based on the literature, it is likely to assume that anesthesia may also have an effect on the cerebrovascular glycocalyx.^69–71^ As such, the PBR values calculated in our study could be slightly elevated because of anesthesia effects. However, this would not explain the difference found between patients and controls, as it should affect PBR values of both groups equally. This warrants further investigation in future studies.

Limitations regarding the fluorescent lectin staining relate to the fact that only four patients’ tissue samples were stained adequately for comparison with *in vivo* results. Staining results were insufficient in five patients’ tissue, possibly due to differences in tissue quality and timing. Additionally, *ex vivo* results were evaluated in slices that were 5 µm thick, rendering it impossible to visualize a ‘continuous network’ of blood vessels, as is performed in our *in vivo* method. Further histological vascular density analysis of thicker slices (30–50 µm) might provide this result, preferably using 3 D visualization such as two-photon microscopy imaging.^72^

### Future perspectives

Only 20–50% of patients with drug-resistant epilepsy are surgical candidates. Possible new therapies are therefore desperately needed to allow improved treatment for this group. In this regard, it is noteworthy that most microcirculatory abnormalities were found in the cortex of TLE patients, but not in the hippocampal microcirculation. The high hippocampal recruitment capacity might indicate a greater role of the temporal neocortex in TLE than previously thought, as abnormalities described in TLE are often linked to the hippocampus, though no control data was available for data obtained in the hippocampal microcirculation. The abnormalities found in the neocortex might suggest that these are also present in the pathophysiology of epilepsy in general, not just in TLE. Indeed, studies have found increasing evidence that epilepsy is accompanied by BBB dysfunction in the neocortex.^12,42,43^ As such, further research should be aimed at confirming glycocalyx shedding and microcirculatory abnormalities in epilepsy. Recently, attempts have been made to use shedded glycocalyx elements as biomarkers for central nervous system diseases.^73,74^ Circulating heparan sulfates have been shown to selectively penetrate hippocampal tissue causing functional disturbances in mice, contributing to epileptogenesis.^75^ Morphological changes, as well as detection of shedded glycocalyx components and composition alterations must be further investigated to gain a clearer picture of brain pathologies and healthy brains. Similarly, *in vivo* assessment of BBB dysfunction and microcirculatory properties requires further evaluation. A potential, less invasive approach towards this would be the use of ultra-high field MRI and sequences such as blood oxygenation level dependent functional MRI (BOLD fMRI).^76,77^ Since glycocalyx disintegration is present in TLE, therapies that regulate BBB integrity may represent a robust way of treating patients who become drug-resistant to the current standard of care. Targeted therapy to restore the glycocalyx may present a new generation of drugs to prevent epileptogenesis or treat epilepsy and other neurological conditions where BBB breakdown is a hallmark pathology.^55^ Several studies have explored the potential of prevention and restoration of BBB integrity and glycocalyx shedding in laboratory and preclinical conditions. Targets such as claudin-5, sphingosine-1 phosphate, or a disintegrin and metalloproteinase 15 (ADAM 15) may be interesting, as well as the use of liposomal nanocarriers to restore a degraded glycocalyx.^39,44,45,78^ Based on our findings, the next logical step will be to assess the therapeutic efficacy of restoring the glycocalyx and microcirculatory dysfunction in epilepsy models, which might ultimately be deployed clinically.

In conclusion, this is the first clinical study to evaluate the structural and functional properties of cerebral microcirculation *in vivo*. Our findings show that the cortical microvasculature of patients with TLE is characterized by reduced glycocalyx integrity. Moreover, patients with TLE have impaired cortical blood flow control and capillary recruitment capacity. *In vivo* and *ex vivo* blood vessel surface area quantification was strongly correlated, highlighting the reliability of *in vivo* data. Further assessment of cerebral microcirculation and its glycocalyx in relation to epileptogenesis is needed to elucidate their role in the pathophysiology of epilepsy. As such, the glycocalyx and microcirculation may form a potential novel target in the treatment of drug-resistant epilepsy.

## The following are members of the ACE epilepsy surgery group

Gwendolyn de Bruyn, Albert Colon, Jim T.A. Dings, Marc Hendriks, Danny Hilkman, Christianne Hoeberigs, Jochem van der Pol, Lotte de Jong, Kim Rijkers, Sylvia Klinkenberg, Vivianne van Kranen – Mastenbroek, Jeske Nelissen, Pieter Kubben, Walter M. Palm, Paul Hofman, Rob P.W. Rouhl, Olaf E.M.G. Schijns, Simon Tousseyn, Marielle Vlooswijk, Louis Wagner, Dorien Weckhuysen, and Guido Widman.

## Consent to participate

Informed consent was obtained from all individual participants included in the study.

## Consent to publish

The authors affirm that human research participants provided informed consent for publication of their data and images.

## Supplemental Material

sj-pdf-1-jcb-10.1177_0271678X231179413 - Supplemental material for Cerebrovascular glycocalyx damage and microcirculation impairment in patients with temporal lobe epilepsy Click here for additional data file. Supplemental material, sj-pdf-1-jcb-10.1177_0271678X231179413 for Cerebrovascular glycocalyx damage and microcirculation impairment in patients with temporal lobe epilepsy by Rick HGJ van Lanen, Roel HL Haeren, Julie Staals, Jim TA Dings, Olaf EMG Schijns, Govert Hoogland, Sander MJ van Kuijk, Dimitris Kapsokalyvas, Marc AMJ van Zandvoort, Hans Vink and Kim Rijkers in Journal of Cerebral Blood Flow & Metabolism
