# Supplementary material for: Cerebrovascular glycocalyx damage and microcirculation impairment in patients with temporal lobe epilepsy
Source: J Cereb Blood Flow Metab. 2023 May 26;43(10):1737–51. doi: 10.1177/0271678X231179413 (PMC10581235; doi:10.1177/0271678X231179413)
Supplement: sj-pdf-1-jcb-10.1177_0271678X231179413 - Supplemental material for Cerebrovascular glycocalyx damage and microcirculation impairment in patients with temporal lobe epilepsy [file sj-pdf-1-jcb-10.1177_0271678X231179413.pdf]

Supplementary material

Supplementary material 1

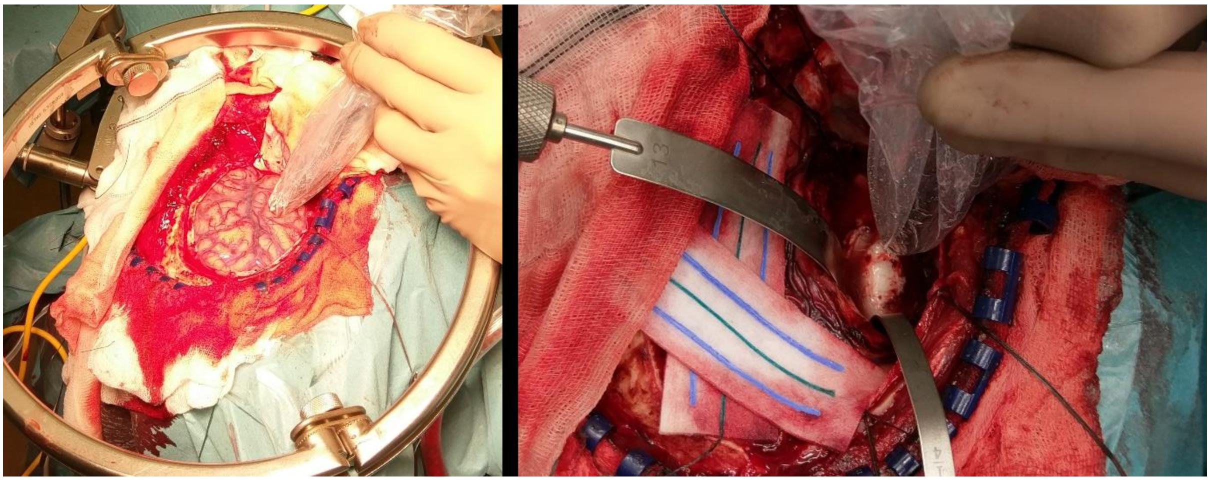

Supplemental figure 1. Intraoperative measurements. Left: cortical measurement directly on the cerebral cortex using the SDF imaging video microscope with a sterile slipcover. Right: hippocampal measurement performed upon removal of the temporal neocortex, allowing a lateral view of the hippocampus. (A license to reuse this figure from its original source was acquired through RightsLink. Haeren et al., Journal of Neuroscience Methods, 2018.(28))

Supplementary material 2

As stated in the results, a total of 41 SDF microcirculation measurements were completed (27 cortical and 14 hippocampal measurements). During a recording, GlycoCheck software automatically selects vascular segments in each video, and continues to acquire data until 3,000 vascular segments are captured. A complete acquisition usually contained 10-30 videos, depending on the number of blood vessels that could be captured by the camera per recording site. GlycoCheck has a built-in quality control in which the software checks all selected vessel segments. These vessel segments are only analyzed further if: (1) red blood cell content in a selected vessel segment is ≥ 50%, (2) red blood cell column width is ≥ 2μm, (3) the segment is not curved, or (4) segments are too close to each other, causing overlapping vessel segments.[29] To validate this built-in quality control, we performed manual verification of all measurements. All individual videos captured by the SDF camera were assessed independently by two reviewers (RHGJvL and RHLH) after all measurements were completed. In case of disagreement between reviewers on whether to include a video for further analysis (three occasions), a third reviewer (KR) made the final decision to in/exclude the video.

This manual assessment identified 28 measurements (18 cortical, 10 hippocampal) for which we excluded one or more videos. Reasons for poor video quality and vessel segment selection were the presence of air bubbles, light artefacts, blurry images, or measurement of extravascular erythrocytes by the SDF camera. Poor video quality was most likely a consequence of the sterile slipcover and gel used for the measurements. Additionally, the physiological pulsations of the brain, along with the presence of cerebrospinal fluid around the hippocampus occasionally hampered the camera's focus. These effects have been identified before.[28] Supplementary fig. 1 shows an example of a sufficient (A) and an insufficient (B) image quality, of which the latter was excluded from further analysis.

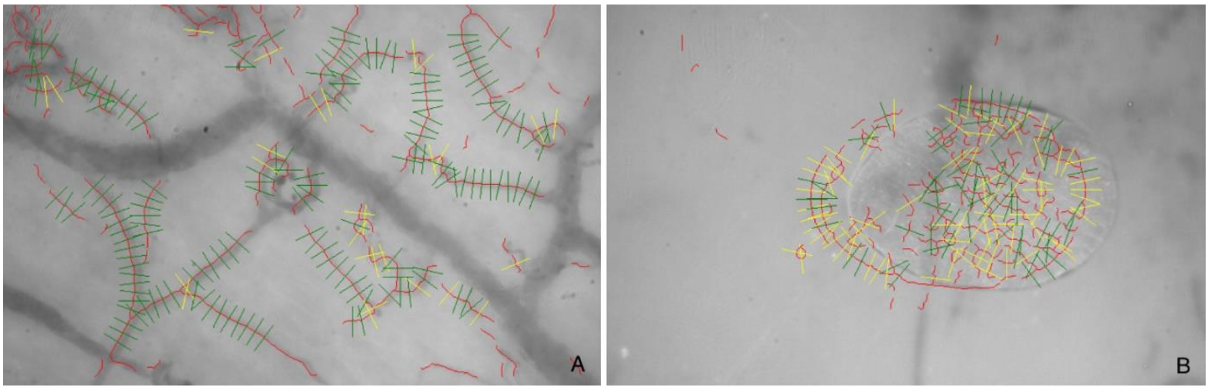

Supplemental figure 2. Photomicrographic stills of an intraoperative SDF recording. Images showing blood vessels as automatically detected by GlycoCheck software. Red lines indicate an identified blood vessel, green lines a valid blood vessel segment for measurement, yellow lines an invalid measurement. **(A)** example of an included video, showing correct blood vessel and segment detection. **(B)** example of a video showing an air bubble obstructing the SDF camera from adequately visualizing the microvasculature, which was therefore excluded from further analysis.

Manual selection resulted in the loss of a portion of possible data (average valid vessel segments: controls cortical 1982/3000=66.1%; patients cortical 1444/3000=48.1%; patients hippocampal 1477/3000=49.2%). We compared PBR values of manually selected videos with vessel segments automatically selected by GlycoCheck software. In the cortex of controls, this comparison showed a similar PBR between manual and automatic selection (1.26 µm (SD 0.23) vs. 1.31 µm (SD 0.29), *P*=0.96). For TLE patients, the cortical PBR was also similar (2.70 µm (SD 0.31) vs. 2.64 µm (SD 0.52), *P*=0.97), as was the hippocampal PBR (2.44 µm (SD 0.22) vs. 2.53 µm (SD 0.39), *P*=0.94). Since no difference was found, we validated that GlycoCheck software automatically discards vessel segments within each video that do not meet the criteria required for further analysis.

Of the videos that were excluded from further analysis after manual identification of insufficient quality, many still had several vessel segments there were adequately visualized. Similarly, manually selected videos that were included for further analysis still had some incorrect vessel segments that should be discarded. By using automatic GlycoCheck vessel segment selection, the correct vessel segments are adequately selected for further analysis,

while the incorrect vessel segments in each video are discarded. Therefore, we decided to use the data generated after automatic selection of correct vessel segments by GlycoCheck software. This automatic selection resulted in the loss of a portion of possible data (average valid vessel segments: controls cortical 1369/3000=45.6%, patients cortical 1044/3000=34.8%; patients hippocampal 1305/3000=43.5%).

Supplementary material 3

Both neocortex and hippocampus tissue samples were collected from 10 patients (P2, P3, P5, P7, P8, P9, P10, P11, P12, P13). Tissue was obtained during surgical procedure; the interval between first tissue manipulation and actual removal of the tissue varied between 15 and 90 minutes, and the time interval between tissue removal and fixation varied between 5 and 30 minutes. These samples were fixed in buffered 4% formaldehyde at room temperature upon resection; after 48 hours of fixation, they were embedded in paraffin. For each patient, one neocortex and one hippocampus cross-section (5 µm in thickness) were deparaffinized, rehydrated using Milli-Q, and incubated first for one hour at room temperature with Ulex Europaeus Agglutinin I lectin (10 µg/mL, UEA I DL-1067; Vector Laboratories USA), then for 20 minutes at room temperature with DAPI (1:10,000; Roche Diagnostics, Switzerland). After incubation, tissues were thoroughly washed five times with 200 µL TBS-T for five minutes each, and mounted using ProLong Diamond Antifade (P36961; ThermoFisher, USA). The slides were dried at 37°C for five minutes, and stored at 4°C.

Visualization was performed using a fluorescence microscope (Olympus BX51WI) equipped with a digital camera (EM-CCD; C9100; HAMAMATSU PHOTONICS Europe GmbH) using 10x magnification (OPlanSApo; 10x/0.40; ∞/0.17/FN26.5). Red, green and blue filters were used in QuPath (version 0.3.2) to image lectin-stained vessels, lipofuscin and nuclei, respectively.[36] Sensitivity and offset for all filters were zero. Images were scaled and analyzed using FIJI (ImageJ; software version 2.3.0/1.53q).[37] To improve identification of blood vessels, the autofluorescence from lipofuscin was reduced by spectral separation of the red and green filter using the FIJI plugin Poisson NMF.

Total Blood vessel Stained Surface Area (BSSA) was quantified by automated annotation using the red channel in QuPath.[36] Regions with folded tissue were excluded using the ‘wand’ tool.

An appropriate threshold for all samples was created using the ‘pixel classification’ function with Full Resolution (0.81 µm/px), the Gaussian prefilter, and a smoothing sigma of 0.5 for automated quantification. We used a total of three thresholds throughout the data collection to accurately quantify blood vessels and their surface area, as no single appropriate threshold using the ‘pixel classification’ function for automated quantification could be created. BSSA was calculated for neocortex and hippocampus tissue samples by dividing total vessel area (µm^2) by total sample area (µm^2).

Supplementary material 4

Tables 1. Epilepsy characteristics of temporal lobe epilepsy patients

**Patient**	**P1**	**P2**	**P3**	**P4**	**P5**	**P6**	**P7**
Sex	Female	Female	Female	Male	Female	Female	Male
Age (years)	26	46	50	47	53	55	40
Body Mass Index (kg/m^2)	25.6	26.9	25.6	28.1	25.5	22.5	23.3
Smoking	No	No	No	No	No	No	No
Age at first seizure (years)	15	20	1	18	43	40	20
Time since onset of epilepsy (years)	11	26	49	29	10	15	20
Present seizure types*	1, 2, 5	3, 5	1, 2, 3, 5, 7	1, 2, 5	4, 5	1, 5, 7	1, 4
Average seizure frequency	≤ 1 time/month	2-6 times/week	≤ 1 time/2 weeks	≤ 1 time/week	≤ 1 time/month	2-6 times/week	2-6 times/week
Average seizure duration	1-5 minutes	1-5 minutes	1-5 minutes	1-5 minutes	1-5 minutes	1-5 minutes	1-5 minutes
Frequency of status epilepticus	Unknown	Unknown	Unknown	Unknown	Never	≤ 1 time/6 months	Never
Use of antiseizure medication (dose)	Carmabazepine (800mg), levetiracetam (2000mg)	Carmabazepine (800mg), lamotrigine (550mg), rescue medication	Carmabazepine (700mg), levetiracetam (1000mg), clobazam (40mg), zonisamide, rescue medication	Oxcarbazepine (1800mg), phenytoin (300mg)	Carmabazepine (800mg)	Lamotrigine (350mg), clobazam (10mg), rescue medication	Lamotrigine (600mg)

**Patient**	**P8**	**P9**	**P10**	**P11**	**P12**	**P13**	**P14**	**P15**
Sex	Female	Female	Female	Male	Female	Male	Female	Male
Age (years)	33	28	37	42	23	41	52	25
Body Mass Index (kg/m^2)	32.3	22.3	23.3	28.4	23.3	21.7	32.8	27.8
Smoking	No	No	No	No	Yes	No	No	Yes
Age at first seizure	31	25	20	6	18	12	27	19
Time since onset of epilepsy (years)	2	3	17	36	5	29	25	6
Present seizure types*	1, 5, 7	5, 7	4, 5	2, 3, 6, 7	6	5, 7	1, 4, 6	1, 2
Average seizure frequency	> 1 time/day	> 1 time/day	1 time/day	Sometimes 2-3 times/week, sometimes ≤ 1 time/month	≤ 1 time/month	> 1 time/day	2-6 times/week	≤ 1 time/month
Average seizure duration	1-5 minutes	31-60 seconds	≤ 30 seconds	≤ 30 seconds	1-5 minutes	Unknown	1-5 minutes	1-5 minutes
Frequency of status epilepticus	≤ 1 time/6 months	Never	Unknown	Once	Never	Unknown	≤ 1 time/6 months	Never
Use of antiseizure medication (dose)	Carmabazepine (600mg), lamotrigine (300mg), clobazam (5mg), rescue medication	lamotrigine (550mg)	Carmabazepine (1000mg), levetiracetam (2000mg), clobazam (20mg), rescue medication	Levetiracetam (1000mg), lamotrigine (400 mg)	Carmabazepine (900mg), lamotrigine (250mg), clobazam (10mg)	Lamotrigine (250mg), rescue medication	Carmabazepine (1000mg), clobazam (10mg), rescue medication	Lacosamide (400mg), perampanel (10mg), brivaracetam (200mg)

* Seizure types: 1 = aura's, 2 = automatisms, 3 = staring, 4 = simple partial, 5 = complex partial, 6 = generalized tonic-clonic seizure, 7 = secondary generalized tonic-clonic seizure, 0 = other

Medical history of temporal lobe epilepsy patients

**Patient**	**P1**	**P2**	**P3**	**P4**	**P5**	**P6**	**P7**
History of febrile seizures	No	No	Yes, at age 1	No	No	Unknown	No
History of traumatic brain injury	Yes, at age 2. Fall on head with loss of consciousness	Unknown	Unknown	Yes, at age 5. Concussion, trauma unknown	No	No	Yes, at age 3. Fall from stairs
History of cerebral infection	No	Unknown	No	No	No	Unknown	No
History of epilepsy in family	Yes, nieces/ nephews on father's side	Yes, brother of patient	Yes, niece on mother's side	Yes, niece on father's side and sister's daughter	No	Yes, son of patient	No
Medication (non-ASM)	-	levothyroxine, levocetirizine	Irbesartan, metoprolol, desloratadine	-	-	-	-
Medical history	-	Hypothyroidism	Uterus myoma, nasal septum correction	-	Lumbar radicular syndrome, adnexitis	C-section, hepatitis	Migraine, inguinal hernia repair

**Patient**	**P8**	**P9**	**P10**	**P11**	**P12**	**P13**	**P14**	**P15**
History of febrile seizures	Yes, age unknown	Unknown	No	No	No	Unknown	Unknown	No
History of traumatic brain injury	No	No	No	No	No	Yes. Trauma unknown	No	No
History of cerebral infection	No	Yes, meningitis at age 2	No	Yes, meningo-encephalitis at 9 months old	No	No	No	No
History of epilepsy in family	Yes, multiple people on mother's side	No	No	No	Yes, mother of patient	No	No	Yes, paternal grandmother
Other non-ASM drug use	Desloratadine, paracetamol	-	-	-	-	-	Venlafaxine	Pantoprazole, sertraline
Medical history	Jaundice, chronic fatigue syndrome, fibromyalgia, Tietze syndrome	Shoulder operation	Inguinal hernia, eye muscle repair surgery	Tonsillectomy	Infectious mononucleosis	-	Factor V Leiden, pulmonary embolism, DVT	SEEG implantation

MRI, (S)EEG and histopathological findings of temporal lobe epilepsy patients.

Patient	P1	P2	P3	P4	P5	P6	P7
Side of epileptic focus	Right	Left	Right	Right	Left	Right	Right
Hippocampal sclerosis on MRI	Yes, right side	No	Yes, right side	No	No	No	No
Focal cortical dysplasia on MRI	No	No	No	No	Yes, left side*	No	No
Ictal onset zone on (S)EEG	Neocortical TLE (NTLE)	NTLE	NTLE	Mesial TLE (MTLE)	MTLE	MTLE	NTLE
**Findings during histopathological examination**							
Hippocampal sclerosis (HS)	Yes	Yes	Yes	No	No	Could not be determined	No
HS according to Blümcke	Type 1	Type 2	Type 1	-	-	-	-
HS according to Wyler	Could not be determined	Could not be determined	Could not be determined	-	-	-	-
Focal cortical dysplasia (FCD)	No	No	No	No	No	No	No

Patient	P8	P9	P10	P11	P12	P13	P14	P15
Side of epileptic focus	Right	Left	Right	Left	Right	Right	Right	Right
Hippocampal sclerosis on MRI	No	Yes, left side	No	Yes, left side	No	No	Yes, right side	No
Focal cortical dysplasia on MRI	No	No	No	No	No	No	No	No
Ictal onset zone on (S)EEG	MTLE	NTLE	MTLE	MTLE	MTLE	MTLE	MTLE	MTLE
**Findings during histopathological examination**								
Hippocampal sclerosis (HS)	Yes	Yes	No	Yes	Yes	No	Yes	No
HS according to Blümcke	Type 1	Type 1	-	Type 1	Type 3	-	Type 1	-
HS according to Wyler	Could not be determined	Grade 3	-	Could not be determined	Grade 3	-	Grade 4	-
Focal cortical dysplasia (FCD)	No	No	No	No	No	No	No	No

Tables 2. Characteristics of control subjects

**Control subject**	**C1**	**C2**	**C3**	**C4**	**C5**	**C6**	**C7**
Sex	Female	Male	Male	Female	Female	Female	Male
Age (years)	52	50	55	49	54	46	53
Body Mass Index (kg/m^2)	32.7	25.3	27.7	19.6	36.4	34.1	33.2
Smoking	No	Yes	No	Yes	No	Yes	No
Indication for surgery	Tumor	Vascular	Tumor	Vascular	Tumor	Vascular	Tumor
Diagnosis	Meningioma	Aneurysm of left middle cerebral artery	Adenocarcinoma nasal cavity with skull base and dural infiltration	Aneurysm of right middle cerebral artery	Meningioma	Aneurysm of right middle cerebral artery	High grade glioma
Side and location of surgery	Right frontal	Left temporal	Bilateral frontal	Right temporal	Left occipital	Right temporal	Right parietal
Abnormal findings during neurological examination	None	Blindness left eye. Loss of strength and sensibility lower extremities	None	None	Hemianopia	Paralysis and loss of sensibility in lower extremities	None
Medication	Quetiapine	Fluticasone/ salmeterol	-	Temazepam	Pantoprazol	Gabapentine, morphine, duloxetine, pramipexol, salbutamol, mebeverine, triamcinolone, azelastine/fluticason	Naproxen, omeprazol, dexamethason
Medical history	-	Inguinal hernia repair, ear operation	-	-	Cholecystectomy, knee replacement, breast reduction, eardrum surgery	Carpal tunnel release, fibromyalgia, IBS, uterus extirpation, chronic backpain	Gout, pericarditis, bicuspid aortic valve, neuropathy, partial meniscectomy
History of traumatic brain injury	No	Yes, scooter accident	No	No	Unknown	No	No

Control subject	C8	C9	C10	C11	C12	C13	C14	C15
Sex	Male	Female	Male	Male	Female	Female	Male	Male
Age (years)	59	59	49	52	32	44	59	23
Body Mass Index (kg/m2)	30.4	26.2	21.4	23.0	32.3	24.7	43.1	24.1
Smoking	No	No	No	No	No	No	No	No
Indication for surgery	Tumor	Vascular	Vascular	Tumor	Tumor	Tumor	Tumor	Tumor
Diagnosis	Meningioma	Aneurysm of left middle cerebral artery	Aneurysm of left middle cerebral artery	Sinonasal carcinoma	Low grade glioma	Low grade glioma	High grade glioma	Ependymoma
Side and location of surgery	Right frontal	Left frontal	Left temporal	Bilateral frontal	Right temporal	Right parietal	Left frontal	Left frontal
Abnormal findings during neurological examination	None	None	None	Diplopia	Hypesthesia left side of face. Slight central facial nerve palsy on left side	None	Abnormal tandem gait	Babinski reflex of right foot
Medication	Clorazepine acid, colchicine, venlafaxine	Omeprazol, letrozole	Lercanidipine	-	-	-	Cholecalciferol, dexamethason	Dexamethason, pantoprazol
Medical history	Prostate cancer	Mamma carcinoma, cervical carcinoma, pulmonary embolism, renal embolism	Polycystic kidney disease, kidney dysfunction	Bicuspid aortic valve, coarctation of aorta	Cervical dysplasia	BPPV	Obstructive sleep apnea	-
History of traumatic brain injury	Yes, car accident	No	No	No	No	No	No	No

Table 3. Intra-operatively recorded parameters at time of measurement.

	**Patients**	**Controls**	p-value
Systolic blood pressure (SD), mmHg	104.47 (15.88)	109.27 (14.84)	.436
Diastolic blood pressure (SD), mmHg	59.33 (11.98)	65.27 (12.20)	.190
Heartrate (SD), bpm	70.33 (12.36)	66.73 (10.11)	.390
Oxygen saturation (SD), %	98.60 (1.35)	98.93 (1.10)	.539
End-tidal CO2 (SD), kPa	4.34 (0.50)	4.37 (0.38)	.852
Hemoglobin level (SD), mmol/L	8.71 (1.03)	8.93 (0.92)	.661
Hematocrit level (SD), L/L	0.43 (0.04)	0.43 (0.05)	.956

Supplementary material 5

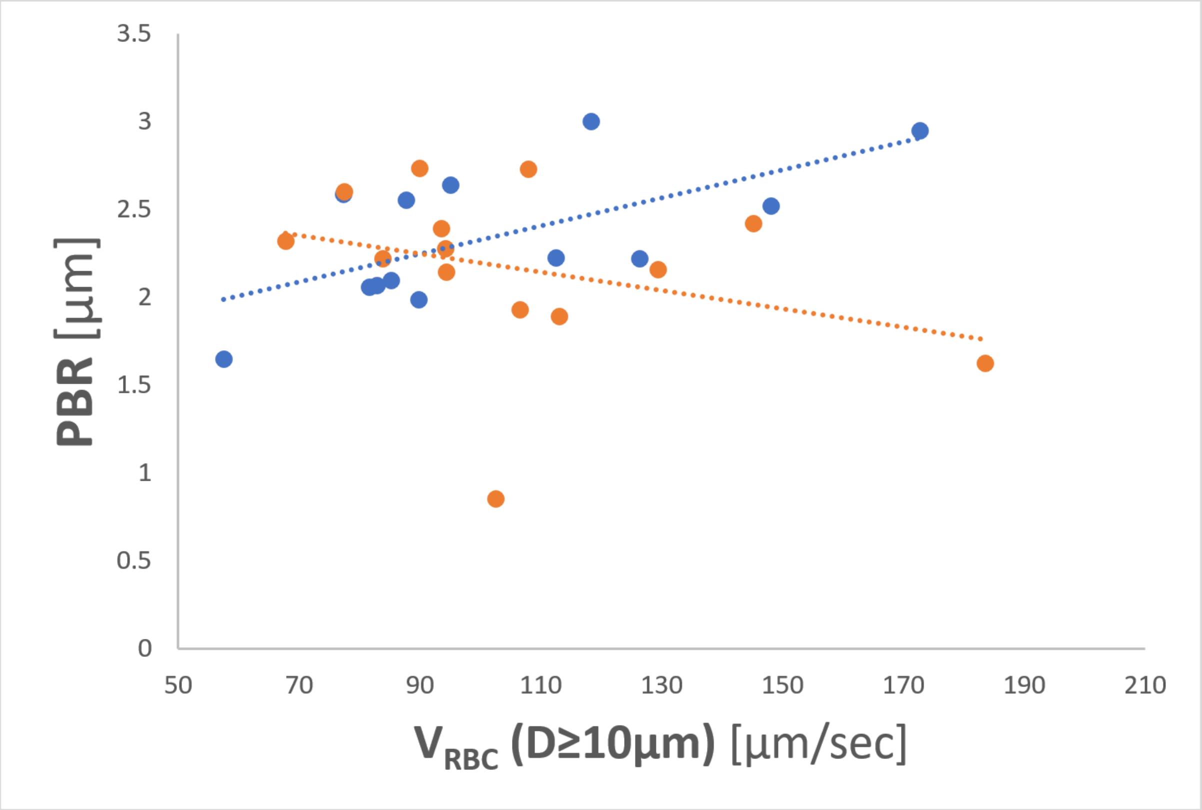

Supplemental figure 3. Flow dependency of perfused boundary region (PBR) in feed blood vessels. Controls show a positive slope (low PBR at low flow sites, and high PBR at high flow sites, indicating that metabolic flow control is intact, and that low metabolic tissue sites have low blood flow and low PBR due to low metabolic challenge of microvascular glycocalyx. In contrast, patients show a negative slope (high PBR at low flow sites), indicating impaired metabolic flow control; low flow site is the site of high metabolic demand (flow-demand mismatch).
